# An improved protein structure evaluation using a semi-empirically derived structure property

**DOI:** 10.1186/s12900-018-0097-0

**Published:** 2018-12-12

**Authors:** Manoj Kumar Pal, Tapobrata Lahiri, Garima Tanwar, Rajnish Kumar

**Affiliations:** Department of Applied Science, Indian Institute of Information Technology, Biomedical Informatics Lab, Room no 4302, CC2 Building, Allahabad, UP 211012 India

**Keywords:** Protein structure validation, Semi-empirical method, Surface roughness index, Confusion set of models, Selection of best structure model

## Abstract

**Background:**

In the backdrop of challenge to obtain a protein structure under the known limitations of both experimental and theoretical techniques, the need of a fast as well as accurate protein structure evaluation method still exists to substantially reduce a huge gap between number of known sequences and structures. Among currently practiced theoretical techniques, homology modelling backed by molecular dynamics based optimization appears to be the most popular one. However it suffers from contradictory indications of different validation parameters generated from a set of protein models which are predicted against a particular target protein. For example, in one model Ramachandran Score may be quite high making it acceptable, whereas, its potential energy may not be very low making it unacceptable and vice versa. Towards resolving this problem, the main objective of this study was fixed as to utilize a simple experimentally derived output, Surface Roughness Index of concerned protein of unknown structure as an intervening agent that could be obtained using ordinary microscopic images of heat denatured aggregates of the same protein.

**Result:**

It was intriguing to observe that direct experimental knowledge of the concerned protein, however simple it may be, might give insight on acceptability of its particular structural model out of a confusion set of models generated from database driven comparative technique for structure prediction. The result obtained from a widely varying structural class of proteins indicated that speed of protein structure evaluation can be further enhanced without compromising with accuracy by recruiting simple experimental output.

**Conclusion:**

In this work, a semi-empirical methodological approach was provided for improving protein structure evaluation. It showed that, once structure models of a protein were obtained through homology technique, the problem of selection of a best model out of a confusion set of Pareto-optimal structures could be resolved by employing a structure agent directly obtainable through experiment with the same protein as experimental ingredient. Overall, in the backdrop of getting a reasonably accurate protein structure of pathogens causing epidemics or biological warfare, such approach could be of use as a plausible solution for fast drug design.

## Background

Development of method to provide fast solution of protein structure is important for many reasons. First off, important roles of proteins particularly in living systems, such as, regulating, catalyzing, and triggering many biological, immunological as well as pathophysiological processes. This has led to development of both experimental and theoretical prediction methods to accomplice this task. However, experimental methods are constrained with want of protein crystals for X-Ray Crystallography; smaller proteins (< 80 KD) for NMR and lengthy experimental time (nearly 2 years) for both of these methods [1, 2]. On the other hand, theoretical prediction methods are although quite fast, suffers limitation of less accuracy and reliability [3]. This creates an apparently unbridgeable huge gap between number of proteins and their known structures [4]. The most popular theoretical prediction method, homology modelling builds initial structure model through comparison of similar templates searched out from database of known protein structures [5–8]. The initial model is subsequently optimized through Molecular Dynamics Simulation producing a set of Pareto-optimal candidate structure models. The trouble starts from there due to confusion posed by contradictory indication of different validation parameters for different models of the same target protein. For example say, Ramachandran score of a model is quite high while its potential energy is not that low and vice versa. The complexity further increases due to addition of more important validation parameters, such as, G-factor which gives account of model’s adherence to steric hindrance property, Verified 3D providing insight to compatibility of an model with its own amino acid sequence, etc.. Drawbacks of knowledge based validation parameters used for acceptability of protein structure are well reported in the review of Kihara et al. [9]. For this reason, functional utilization of structure models outputted by comparative prediction methods is very challenging [9]. This piece of work put effort to resolve this problem through the intervention of simple experimental output obtainable from the target protein. The idea is that a protein of known sequence can be isolated, purified into a sizeable quantity to perform many simple experiments on it. Examples of such experiments are: study on patterns of temperature-function kinetics, pH-function kinetics in presence of substrates, aggregation through heat denaturation, emission-absorption spectra etc.. It appears to be interesting to see whether any of such experimental output can be utilized to select best structure model out of a set of such models resulted in through theoretical exercise. Furthermore, to corroborate correctness of theoretically found models through such experimentally found information there must be a common parameter that can be extracted both from the experimental information as well as from the structures. Only then, this common parameter extracted from experiment can be stored and utilized as standard to compare its closeness with that extracted from predicted structure models for picking the closest one as the best structure model. Also, this common parameter should also have the attribute of uniqueness at least for the target protein class if not for the actual protein itself. In search of such parameter, in this study first it was identified that Surface Roughness Index (SRI) of a protein as derived, calculated from its known structure by Singha et al. [10] might be utilized as common structure parameter since it could also be extracted through experiment on the same protein as depicted by Mishra et al. [11]. In this regard the role of predicted SRI was to serve as a standard parameter that can be compared for its closeness with the values calculated from the predicted models to pick the best structure solution under the premise: closest model was the best one. Finally this semi-empirical structure validation method was tested for some judiciously chosen proteins taken from protein data bank (PDB) of widely varying structure class which could also be procured through purchase for further experimentation. To test whether the method could match the real life challenge for protein structure evaluation, the template search step of Homology Modelling was specifically employed to select first three templates with sequence similarities less than equal to 77% applying BLASTP. The cut-off 77% was chosen considering the fact that a sequence similarity more than 90% guaranteed to produce structure comparable to X-Ray crystallographic structure of a protein except for a few individual side chains [17–19] thus making this study unnecessary. Also, the first hit having 100% similarity was intentionally ignored since this was the target protein itself and already present in PDB. In the next step, for the output structure models generated through Homology Modelling, different knowledge based validation parameters were calculated. As expected contradictory indications from these validation parameters generated a confusion set of structure models the correctness of which was finally resolved by the use of experimentally extracted value of SRI. The final validation of the selected model was done by comparing root mean square deviation (RMSD) of backbones of all models with that of reported PDB structure of the target protein.

## Methods

### Description of proteins used in this study

Six proteins, albumin, cytochrome c, ferritin, lysozyme, insulin and hemoglobin which could be procured through purchase from the market as well as reported in the PDB site were selected and finally purchased from Sigma Aldrich (USA). Also, these proteins were chosen for experimentation for their widely varying structural properties where the structural properties, class, fold, super family, family, duplication and species as retrieved for these proteins from SCOP [20] were given in the following Table 1. Sample size of such proteins were in concurrence with Sandelowski, 1995 [21].

**Table 1:** Widely varying structural classes of the proteins selected for experimentation

PDB id	Protein name	Class	Fold	Super family	Family	Duplication	Species
1ao6	Serum albumin	All alpha proteins	Serum albumin-like multihelical; one domain consists of two similar disulfide-linked subdomains	Serum albumin link to SUPERFAMILY database - Superfamily	Serum albumin	consists of three domains of this fold	Human (*Homo sapiens*)
1new	Cytochrome c7 (cytochrome c551.5, PpcA)contains three heme groups; deletion of one of Cyt c3 heme-binding sites	All alpha proteins	Multiheme cytochromes variable number of helices and little beta structure; not a true fold	Multiheme cytochromes	Cytochrome c3	contains multiple CxxCH motifs link to SUPERFAMILY	Desulfuromonas acetoxidans
1ro3	Echistatin	Small proteins Usually dominated by metal ligand, heme, and/or disulfide bridges	Blood coagulation inhibitor (disintegrin) small disulfide-rich	Blood coagulation inhibitor (disintegrin) link to SUPERFAMILY database - Superfamily	Blood coagulation inhibitor (disintegrin)	Not Reported	Saw-scaled viper (*Echis carinatus*)
2vb1	Lysozyme ubiquitous in a variety of tissues and secretions	Alpha and beta proteins Mainly antiparallel beta sheets (segregated alpha and beta regions)	Lysozyme-like common alpha+beta motif for the active site region	Lysozyme-like Superfamily	C-type lysozyme	Not Reported	Chicken (*Gallus gallus*)
2h8b	Insulin from Human (in absence of report for 2h8b other columns were filled in for 1ben)	Small proteins (hormone)	Insulin-like nearly all-alpha can be classified as disulfide-rich	Insulin-like link to SUPERFAMILY database - Superfamily	Insulin	Not Reported	Human (Homo sapiens)
1a3n	Hemoglobin, alpha-chain from Human	All alpha proteins	Globin-like core: 6 helices; folded leaf, partly opened	Globin-like link to SUPERFAMILY database - Superfamily	Globins Heme-binding protein	Not Reported	Human (Homo sapiens)

### Obtaining structure models of proteins using homology modelling

To get structure models of a protein Easymodeller Graphical Interface of Kuntal et al. [12] was used to implement Modeller [5–8]. To avoid self-matching with already stored PDB template for the same protein, and also, to avoid templates of very high sequence similarity leading to near perfect solution of structure (as already described in Introduction Section) [17–19], first three templates with sequence similarity obtained through BLASTP ≤77% were chosen as input to Modeller.

### Application of existing validation parameters for selection of best model

Energy Score of a protein was calculated as Random Walk potential following the method of Knowledge Based Scoring Function as described by Zhang and Zhang [13]. However, for calculation of Ramachandran Score and G factor Procheck NT Suite [14] was downloaded from http://www.ruppweb.org/ftp_warning.html and utilized. Verified 3D (% residue) was calculated using Structure Analysis and Verification Server (version 4) following the protocol of Bowie, et al. [15] and Luthy et al. [16]. The justification for choosing these validation parameters was that these parameters were commonly computable for all proteins utilized in this work.

### Calculation of surface roughness index (SRI) of a given protein structure following published protocol

**Fig. 1:**
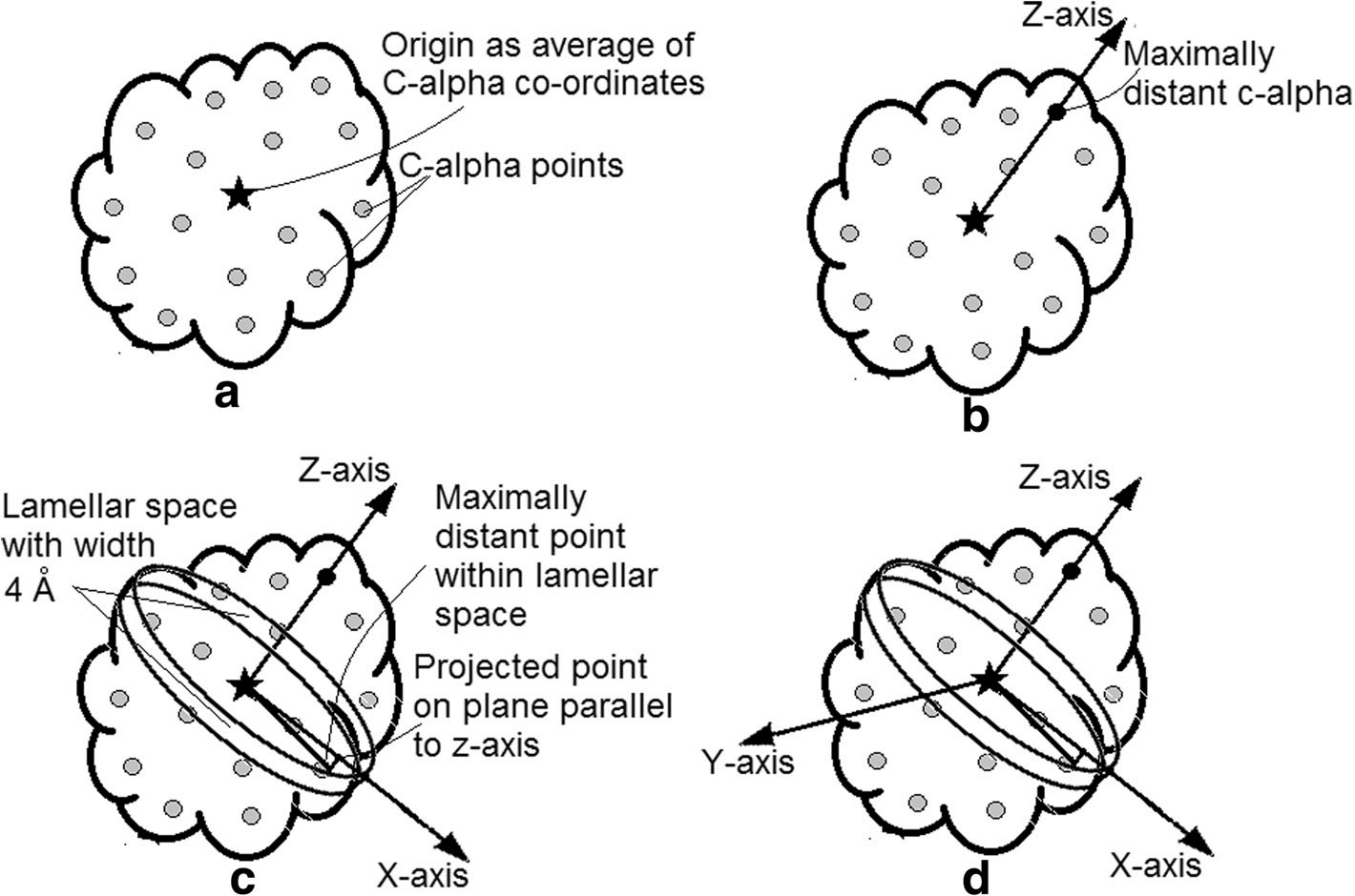
Steps of creation of Invariant Coordinate System (ICS) as described in steps for calculation of SRI: **a**) origin, **b**) Z-axis, **c**) X-axis and **d**) Y-axis

One of the structural component of a protein, its surface was characterized by a 8 valued vector SRI [10]. Each element of this vector can be calculated as standard deviation of distances of its surface residue-points represented by the C-alpha atoms of surface residues falling within a particular octant of a invariant coordinate system (ICS) (described latter) from the protein-centre that is calculated as the average coordinate of all C-alpha coordinates of that protein. In short, as described by Singha et al. [10], ICS of a protein can be calculated following the steps given below:

- Step1: Origin (O) of ICS of a protein is calculated as average coordinate of C-alpha coordinates of all of its residues as shown in (Fig. 1a).
- Step2: Line joining O and maximally distant C-alpha coordinate is considered as z-axis of ICS as shown in (Fig. 1b).
- Step3: Plane normal to z-axis and passing through O is considered as xy-plane. The C-alpha atom which satisfies two constraints: first, it lies within a lamellar space of width 4 Å i.e., within a distance of 2 Å from each side of the xy-plane, and second, its distance from O is maximum among all other C-alpha atoms within this lamellar space, is considered to create x-axis with O as the line joining its projection on xy-plane (P) and O. This step is further clarified in (Fig. 1c).
- Step4: Line passing through O and perpendicular to both x and z-axis is considered as y-axis as shown in (Fig. 1d).
- Step5: The PDB coordinates of all the atoms of a protein are transformed to ICS following simple geometric rule of coordinate transformation.

### Experimental steps in details leading to prediction of SRI following published papers

**Fig. 2:**
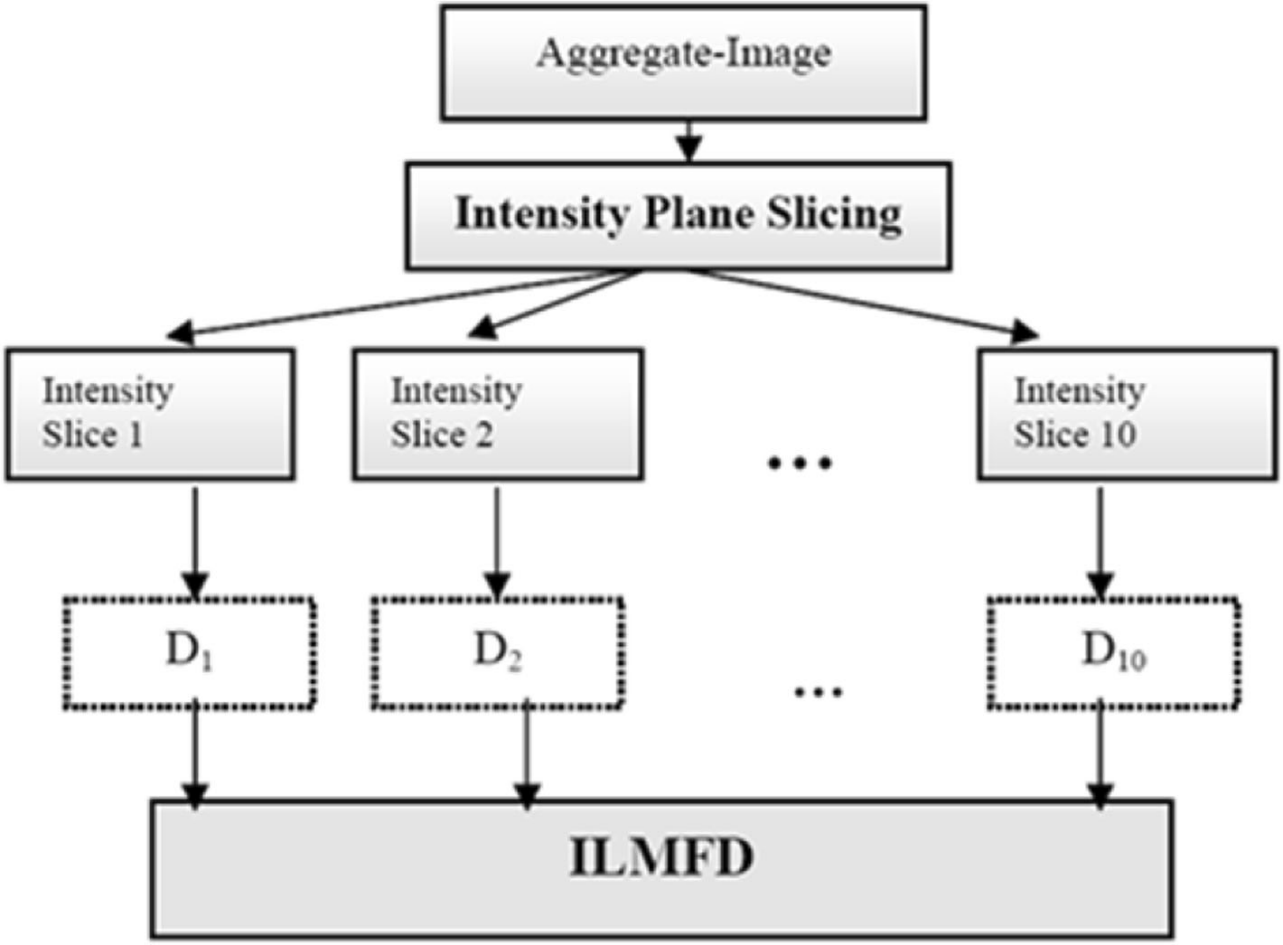
A flowchart depicting extraction of ILMFD parameters from HDA images

**Fig. 3:**
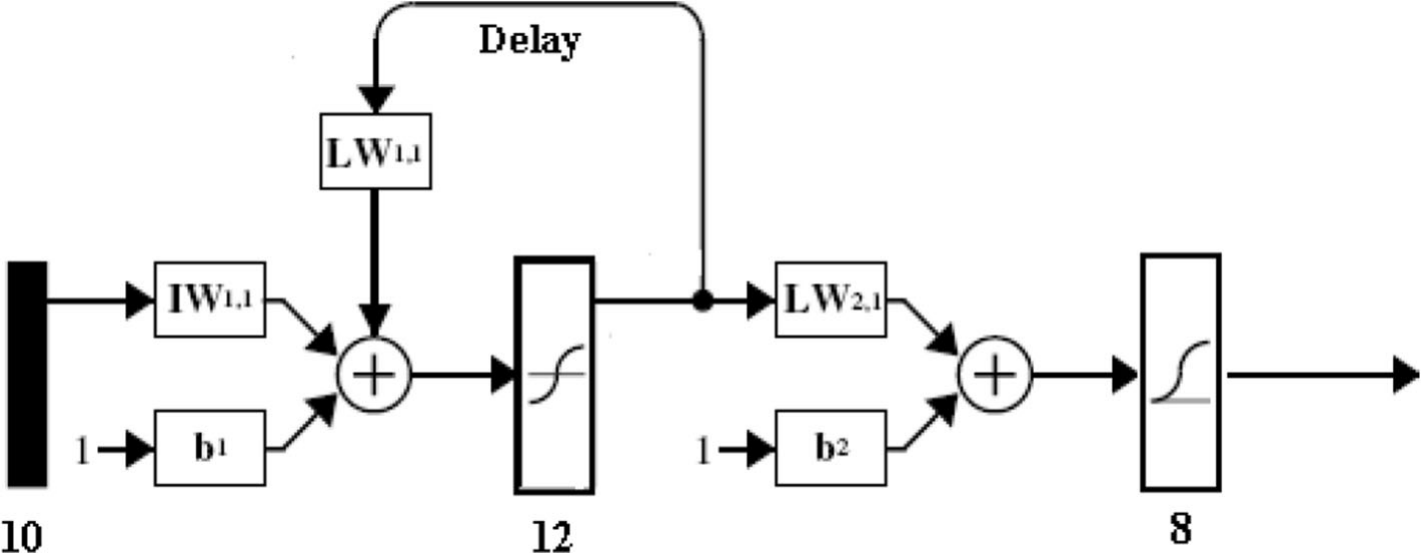
The neural network architecture serving as a non-parametric function to map ILMFD into predicted SRI

SRI of a protein was also predicted through simple experiment from its heat denatured aggregate (HDA) following protocol described by Mishra et al. [11], steps of which were given below:

- Step 1: Solution of the concerned protein was prepared in milipore water at concentration 25 mg/cc and put in hot water bath at temperature 100 °C for 15 min to obtain its HDA.
- Step 2: 10 μL of HDA solution of the protein was put in a hemocytometer slide (Model: Neubauer Chamber, Marienfeld, Germany) and covered with thin microscopic glass cover slip. Subsequently, it was visualized at 400X magnification using phase contrast microscope (Leica Model DML-B2).
- Step 3: Digital images of aggregates were captured using a camera (Canon PowerShot S50) at optical zoom 2X. Thus cumulative optical zoom of the microscope and camera was 800X. 50 images of HDA at different locations of slide were captured for each protein.
- Step 4: Grey scale converted and 1/3rd resized images of HDAs were manually segmented out using MS Paint XP software having intensity range from 0 to 255. Segmented image was further splitted into 10 binary images on the basis of filtering through fixed intensity-ranges by applying the rule described by Mishra et al. [11]. Fractal dimension of each of these binary HDA images were calculated through box-counting method to obtained 10 valued Intensity Level Based Multifractal Dimension (ILMFD). This step is further clarified in (Fig. 2).
- Step 5: A non-parametric function was designed employing Recurrent Backpropagation Neural Network (RBPN) as shown in (Fig. 3) with capability of taking multiple inputs (10 valued ILMFD) and deliver multiple outputs (8 valued SRI after normalization). For optimizing this function 70% of images (i.e., 35 out of 50 images) were used for training and remaining 30% (15 images) were used for testing purpose. For enhancing prediction accuracy through removal of ill-posed noisy data, 15 function-outputs (each of which were 8 valued candidate for predicted SRI) were further passed through a two tier hierarchical clustering method to finally select the centre of the optimally chosen cluster as predicted SRI after denormalization following the algorithm of Mishra et al. [11].

### Steps describing selection of best model using predicted SRI of original protein and calculated SRI of its models

Steps followed to select best structural model of a protein were given below:

- Step1: SRI of each of the structure models of a protein obtained through methodology section 2 were calculated using protocol described in methodology section 4 and designated as SRI_CM. The same method was applied to calculate original protein structure obtained from PDB and was designated as SRI_C.
- Step2: SRI of the original protein was predicted through experiment as described in methodology section 5 and was designated as SRI_P.
- Step3: Euclidean distance, DC_MOD_ between SRI_CM of a model and SRI_C was calculated and it was repeated for all the models. The model with least distance (i.e., that closest to the original) was selected as the best structure model of this protein.
- Step4: Step3 was repeated to calculate distance, DP_MOD_ by replacing SRI_C with SRI_P and the best structure model for the same model was selected.
- Step5: Euclidean distances between coordinates of corresponding C_α_ atoms of a model and original PDB structure were calculated and Root Mean Square Deviation of these distances were stored. Similarly RMSDs for all the models from original structure were calculated. The model with least RMSD was selected for final validation. For calculation of RMSD between a pair of protein structures, coordinates of both of these structures were transformed under Invariant Coordinated System (ICS) as described in methodological section 4.

Step 1 to 5 was repeated for all the 6 proteins used in this study.

## Results

To check coherency in protein structure validation parameters, results of calculation of above-referred validation parameters, Energy Score as Knowledge Based Scoring Function (KBSF), Ramachandran Score (RS), G factor (GF) and Verified 3D (% residue) (V3D) were shown in Table 2 for all the protein models obtained through Modeller along with their PDB structures. However, it was evident from the best matches of the models for a particular protein and validation parameter, the coherency did not exist. For example, in case of albumin, KBSF indicated 2nd model as best, while V3D showed 5th model as the best. Similarly it was shown for all other proteins.

**Table 2:** Different validation parameters obtained for confusion set of models

Name of the proteins	Validation Parameters of models				
	Models	Energy Score	Ramachandran Score	G factor	Verified 3D (% residue)
Albumin	1ao6	− 1.1969 × 10^5^	88.50	0.22	92.39
	1	−1.2280 × 10^5^	95.1	0.16	94.53
	2	−1.2297 × 10^5^	95.4	0.18	96.58
	3	−1.2269 × 10^5^	94.7	0.1	97.09
	4	−1.2262 × 10^5^	94.1	0.17	96.75
	5	−1.2283 × 10^5^	95.2	0.17	99.15
Cytocrome c	1new	−6.6803 × 10^3^	65.50	−0.38	79.41
	1	− 6.3532 × 10^3^	93.1	−0.03	77.94
	2	−6.3340 × 10^3^	91.4	0.00	51.47
	3	−6.3668 × 10^3^	89.7	0.01	79.41
	4	−6.3291 × 10^3^	84.5	0.01	97.06
	5	−6.2220 × 10^3^	89.7	−0.03	73.53
Ferritin	1ro3	−3.4552 × 10^3^	23.7	−0.42	100.00
	1	−4.3944 × 10^3^	76.3	−0.23	93.88
	2	− 4.2660 × 10^3^	78.9	−0.37	100.00
	3	−4.3860 × 10^3^	78.9	−0.21	91.84
	4	−3.7259 × 10^3^	65.8	−0.69	97.96
	5	−3.9113 × 10^3^	63.2	−0.85	100.00
Lysozyme	2vb1	−1.3138 × 10^4^	88.50	0.01	100.00
	1	−2.2767 × 10^4^	93.8	0.06	100.00
	2	−2.2488 × 10^4^	93.8	0.05	100.00
	3	−2.2639 × 10^4^	91.2	0.04	100.00
	4	−2.2616 × 10^4^	94.7	0.08	100.00
	5	−2.2564 × 10^4^	94.7	0.03	100.00
Insulin	2h8b	−7.5931 × 103	77.8	.28	0.00
	1	−4.9314 × 103	84.8	−.16	0.00
	2	−4.9427 × 103	91.3	−.22	0.00
	3	−5.1093 × 103	91.3	−.08	0.00
	4	−4.8367 × 103	80.4	−.15	0.00
	5	−4.8419 × 103	80.4	−.10	0.00
Hemoglobin	1a3n	−1.2495 × 105	94.0	.21	100.00
	1	−1.2209 × 105	91.6	.05	100.00
	2	−1.2216 × 105	91.4	.05	99.31
	3	−1.2295 × 105	91.5	.05	99.31
	4	−1.1820 × 105	88.9	−.26	100.00
	5	−1.2216 × 105	91.4	.05	99.31

The values of SRIs both calculated from known structure and predicted through experiment for the proteins were shown in Table 3 from which parameters DC_MOD_ and DP_MOD_ were calculated.

**Table 3:** SRIs predicted from experiment and calculated from structures for proteins and models

Protein name	Method to obtain SRI	Models	SRI							
Albumin	Predicted through experiment	1ao6	15.10	16.08	22.41	19.45	18.14	9.48	15.04	7.80
	calculated	1ao6	8.22	12.68	12.31	7.70	9.91	9.27	10.09	11.62
		1	9.43	11.42	7.45	12.94	13.06	9.81	7.84	9.92
		2	14.43	11.44	7.11	12.88	11.72	11.68	9.20	9.21
		3	8.80	11.27	7.18	13.46	13.82	10.34	9.35	8.29
		4	11.03	15.31	13.86	8.48	14.31	8.76	7.41	11.17
		5	9.40	11.46	8.55	13.69	13.45	9.64	8.41	9.70
Cytochrome c	Predicted through experiment	1new	5.38	5.83	4.70	2.55	5.11	4.19	3.40	3.70
	calculated	1new	3.45	5.45	4.24	2.02	3.35	2.88	3.47	3.74
		1	4.93	3.77	4.33	1.07	3.11	3.27	3.60	4.07
		2	4.01	0.00	1.83	5.01	3.29	3.40	3.99	3.42
		3	4.41	7.79	1.75	3.77	2.90	3.32	2.13	3.95
		4	3.63	3.58	4.10	1.13	3.66	3.37	4.71	3.97
		5	4.88	4.28	3.37	3.75	2.56	3.07	3.60	4.38
Ferritin	Predicted through experiment	1ro3	4.12	5.23	4.99	3.08	6.33	3.14	3.66	3.23
	calculated	1ro3	0.02	3.20	1.47	0.11	2.14	1.54	2.56	1.77
		1	1.73	3.01	1.38	5.41	3.58	0.61	4.92	1.74
		2	6.31	3.74	5.98	0.90	1.54	4.50	0.00	4.84
		3	2.78	2.66	3.12	4.61	2.77	2.82	3.35	1.37
		4	6.43	0.00	7.66	3.60	0.91	3.39	1.42	2.90
		5	4.15	2.26	1.90	5.25	3.62	1.79	3.25	0.14
Lysozyme	Predicted through experiment	2vb1	3.51	4.29	5.36	4.05	2.84	4.06	4.84	3.93
	calculated	2vb1	4.19	3.32	5.05	5.32	4.23	3.05	2.92	3.83
		1	4.81	4.51	2.77	3.07	4.24	3.12	3.74	3.83
		2	4.06	4.45	2.97	3.30	4.28	2.93	3.63	3.67
		3	3.86	4.47	2.79	3.26	4.51	3.06	3.89	3.84
		4	4.23	4.49	3.84	3.16	4.33	3.01	3.65	3.97
		5	5.81	4.32	2.93	3.03	4.55	2.42	3.76	4.00
Insulin	Predicted through experiment	2h8b	4.59	5.37	5.30	6.23	3.67	4.19	3.51	5.39
	calculated	2h8b	1.66	6.09	4.07	3.96	2.54	3.20	1.92	4.20
		1	2.07	7.54	5.77	2.12	9.05	2.96	3.96	5.67
		2	5.53	4.82	3.14	1.55	8.68	5.22	2.71	1.91
		3	5.75	2.87	2.42	5.13	6.77	3.96	2.11	4.96
		4	7.57	3.27	6.87	2.72	5.49	4.43	1.76	5.99
		5	6.66	3.71	4.77	2.86	1.76	3.98	3.04	5.26
Hemoglobin	Predicted through experiment	1a3n	5.95	8.82	8.59	10.99	9.28	4.96	8.71	8.36
	calculated	1a3n	7.80	8.31	8.69	10.99	8.73	6.31	9.10	6.73
		1	8.85	9.44	9.54	10.29	7.77	9.74	7.95	11.05
		2	9.12	6.62	9.65	8.43	4.89	7.73	8.46	9.05
		3	9.00	5.55	9.52	7.94	4.98	7.69	8.47	8.94
		4	7.09	8.35	8.26	8.80	8.40	6.47	9.16	6.49
		5	9.12	6.62	9.65	8.43	4.89	7.73	8.46	9.05

List of data similar to that shown in Table 2 were prepared using DC_MOD_, DP_MOD_ and RMSD parameters as described in methodology section 6 replacing the validation parameters where best models were obtained using least values of all these parameters. It was intriguing to find that there existed a concurrency of decision provided by all these parameters as shown in Table 4 except for albumin and insulin the explanation for which was given in discussion section.

**Table 4:** Model selection by new validation parameter, predicted SRI

Proteins	Model Selection			
	Best model using DC_MOD_	Best model using DP_MOD_	Best model using RMSD	Mean of RMSD over all models
Albumin	4	4	2	2.94 ± 0.24
Cytochrome C	1	1	1	10.73 ± 6.42
Ferritin	3	3	3	7.90 ± 2.61
Lysozyme	4	4	4	24.31 ± 0.03
Insulin	5	5	2	15.37 ± 2.39
Hemoglobin	4	4	4	29.19 ± 14.70

The diversity in the physiochemical properties and number of residues of the proteins selected for this study was shown in Table 5 below:

**Table 5:** Physiochemical properties and number of residues of selected proteins

Protein name	PDB ID	Average Hydrophobicity considering all the chains	Acidic	Basic	Neutral	No of Residues
Albumin	1ao6	40	16.75	16.92	26.32	585
Cytochrome c	1new	26.47	11.76	26.47	35.29	68
Ferritin	1ro3	20.41	16.33	20.41	42.86	49
Lysozyme	2vb1	34.88	6.98	13.95	44.19	129
Insulin	2h8b	40.20	6.77	13.22	39.83	57
hemoglobin	1a3n	48.10	9.39	16.39	26.12	287

## Discussion

Objective of this study was to strengthen already existing theoretical protocol to expedite solution for protein structure without compromising with accuracy. Towards this direction, published reports were first explored to check whether assimilation of already existing methods can help in achieving the same through development of a new methodical approach. In this regard, the main bottleneck as found in this work was to resolve confusion set of models produced by Homology Modelling with subsequent application of Molecular Dynamics based optimization techniques as reported by [5–8] and implemented through Modeller. However, the confusion in selecting the best model was generated due to incoherent decision provided by different validation parameters as shown in Table 2. As for solution, in this study it was strongly felt to validate those structure models through comparison with a data that can be directly extracted from actual protein of concern through simple experimentation rather than by knowledge based validation parameters, e.g., KBSF, RS, GF and V3D. In this regard, it was imperative to devise a strategy through which model structure could be validated both from the end of the theoretically computable validation parameter as well as that obtainable from actual protein itself, say, through simple experimental exercises as discussed in the Introduction section. It was necessary to see the convergence of validations from both of these ends since in real life problem calculation of SRI values from known protein structure would not be possible and the predicted SRI extracted through experiment was supposed to serve as the only validation agent. It indicated requirement of a common validation parameter which could be obtained both through computational exercise using structure model and experimental method using the same protein as its ingredient. Unfortunately no commonly known existing validation parameters appeared to serve this purpose. However, one such example could be found from the report of Mishra and Lahiri [11] in which a typical structure parameter SRI of a protein was found to be obtained from a semi-empirical method using it as experimental ingredient (as described in methodological section 5) as well as computable from its structure (if known) Singha et al. [10]. As designated in methodological section 6, the need of experimentally extracted parameter SRI_P was to find minimum of DP_MOD_ to pick the best structure model comparing SRI to SRI distances of all the models from SRI_P. Since this methodological approach had to be validated also, only those proteins were selected, PDB structures of which were also available and thus their SRI were also computable (designated as SRI_C) using method of Singha et al. [10] as described in methodological section 4. Therefore, it was left as an interesting exercise to see whether the solution of best model obtained utilizing DP_MOD_ parameter was matching with that obtained utilizing DC_MOD_. Interestingly, while Table 4 showed the result as affirmative for all the six proteins, the final validation of this approach was done by utilizing universally accepted parameter RMSD of corresponding C_α_ to C_α_ distances between a model and already evaluated PDB structure of the protein and repeating it for all the models of the same protein. As shown in Table 4, result of selection of best model using RMSD was further encouraging since it re-confirmed the result using DC_MOD_ and DP_MOD_ except for the cases of Albumin and Insulin. In case of Albumin the possible reason of mismatch might be because of very close proximity of all the models with the original structure as shown in Table 4. Furthermore, in case of Insulin we found the solutions obtained through SRI (i.e., the same 5th model obtained through both DC_MOD_ and DP_MOD_) and RMSD (the 2nd model) are actually closest to each other with lowest RMSD, 4.50. One more interesting observation as found after comparing results of Table 2 and Table 4 was that, percentage of success of RS, GF and V3D in selecting best models through predicted SRI was 50% while that for KBSF was zero only. It indicated that Ramachandran Score, G factor and Verified 3D were better validation parameters in comparison to Energy Score (Knowledge Based Scoring Function). The possible reason of success in SRI based validation as shown in this work for almost all the proteins of wide variety of classes (as shown in Tables 1 and 5) could be explained through its underlying geometric attribute. As designed and implemented by Singha et al. [10] SRI basically represented surface roughness profile of a protein through an 8 valued surface roughness vector each element of which actually represented roughness of protein surface within one out of 8 octants of a 3 dimensional invariant coordinate system containing this protein. They also showed that proteins could be classified at the level of SCOP defined classes by SRI with reasonably high efficiency (almost 85%) which indicated capability of SRI to describe a protein with quite high structural specificity. This attribute of SRI might be considered as the most important factor contributing towards its potential to successfully select best structure model of a protein out of other models. However, SRI being a key agent for such selection, further improvement could be thought of in the design of SRI through creation of optimum number of solid angles as argued by Singha et al. [10] in contrast to 8 in the existing protocol to increase its specificity for a protein. Furthermore, since SRI not only needed to be calculated from a given structure, but also to be extracted from a protein as experimental compound as described by Mishra and Lahiri [11], the root experimental output ILMFD as described above in methodological section 5 might be further looked into to consider its replacement by some other possibly more efficient experimental output e.g., two dimensional excitation-emission spectra of protein suspension within visible light range through simple spectrophotometry instead of Heat Denatured Aggregates as described earlier.

## Conclusions

This work showed a way which could be of help towards fast solution of a protein structure without compromising with its accuracy. The importance of this work was that it provided a methodological approach through which once structure models of a protein were obtained through currently best theoretical exercise, say, Homology Modelling, the problem of selection of a best model out of a confusion set of same could be resolved by employing a structure agent Surface Roughness Index which could be directly obtained through a semi-empirical method using microscopic images of Heat Denatured Aggregates of the same protein as experimental ingredient. Overall, in this work it was emphasized that in absence of an ordered aggregate of protein as its crystal, experimental use of its irregular assemblies could also be of help in solving its structure. In the backdrop of getting a reasonably accurate protein structure of pathogens causing epidemics or biological warfare, such approach could be of use as a plausible solution for fast drug design to contain their effect.

## Abbreviations

GF: G factor
HDA: Heat denatured aggregate
ICS: Invariant coordinate system
ILMFD: Intensity Level Based Multifractal
KBSF: Knowledge Based Scoring Function
PDB: protein data bank
RMSD: Root mean square deviation
RS: Ramachandran Score
SCOP: Structural Classification of Proteins
SRI: Surface Roughness Index
V3D: Verified 3D (% residue)
